# Using the ADDIE Model to Develop a Rusnani Concept Mapping Guideline for Nursing Students

**DOI:** 10.21315/mjms2020.27.6.11

**Published:** 2020-12-29

**Authors:** Rusnani Ab Latif, Mohd Zarawi Mat Nor

**Affiliations:** 1 Universiti Teknologi MARA, Pulau Pinang, Malaysia; 2 School of Medical Sciences, Universiti Sains Malaysia, Kelantan, Malaysia

**Keywords:** development, ADDIE model, Rusnani concept mapping (RCM), academic performance, nursing students

## Abstract

**Background:**

Concept mapping has been established as a learning strategy that encourages critical thinking and creativity among students, leading to the development of a concept mapping guideline designed to guide nurse educators in using this teaching strategy.

**Objectives:**

This paper illustrates the development of a guideline to build a concept mapping based-learning strategy. Called the Rusnani concept mapping (RCM) protocol guideline, it was adapted from the Mohd Afifi learning model (MoAFF) and the analysis, design, development, implementation and evaluation (ADDIE) model, integrated with the Kemp model.

**Methods:**

This model uses the five phases of analysis, design, development, implementation and evaluation. The validity of the guideline was determined by using content and face validity and the Delphi technique. Content validity for this RCM guideline was established using expert review. This formula suggested that if the content validity is greater than 70%, it shows good content validity, and if it is less than 70%, the content validity is low and it is advisable to recheck the content according to the objective of the study.

**Results:**

The reliability of the RCM was 0.816, showing that the RCM guideline has high reliability and validity.

**Conclusion:**

It is practical and acceptable for nurse educators to apply RCM as an effective and innovative teaching method to enhance the academic performance of their nursing students.

## Introduction

Much has been discussed in the literature about the benefits of concept mapping. It has been argued that it is one of the more effective learning strategies requiring students to employ critical thinking (1). In this regard, nursing education is not an exception, where concept mapping is used by students to enhance their understanding of and skill with interrelating and linking information. Producing a good concept map requires critical and analytical thinking. Most of the concept mapping articles found in the body of Western literature expound on the process of creating concept maps or using concept maps in teaching nursing students (2). However, in the Malaysian nursing field, there are only a few studies of concept mapping in a local context, making it difficult to understand how well concept mapping might aid the academic performance of Malaysian nursing students.

Scholars argue that the traditional didactic teacher-oriented approach seems too outdated for the current practices of the nursing profession (3, 4). The new challenges in health care fields demand an instruction strategy that encourages good clinical judgement, problem-solving abilities and nursing care skills (3). The current approach makes long-term learning less likely to occur, primarily because the teacher plays the active part, while students act as listeners and passive recipients. This scenario was partially caused by the urge to finish up the syllabus to cater to exam-oriented traditional teaching approaches.

Critical thinking is an essential component of nursing education if nurses are to deliver safe quality care. It is the role of nurse educators to inculcate the capability for critical thinking in students through suitable learning activities. A failure to do so jeopardises students’ opportunities to develop their critical thinking skills. To that end, the aim of this study was to develop an instructional strategy for nurse educators using Rusnani concept mapping (RCM). The researchers sought to develop and validate the RCM guideline based on the analysis, design, development, implementation and evaluation (ADDIE) model. This article elaborates on the design and development process of the RCM ADDIE model.

## Background

ADDIE is widely used by instruction designers to produce modules, models, software and courseware for instruction and learning (5). It is also employed as a teaching design model that presents a series of repetitive steps to build effective education and training in five phases, which create the acronym: A-D-D-I-E stands for analysis, design, development, implementation, and evaluation (6, 7).

By incorporating the ADDIE model, the objective of RCM is twofold — to improve the educator’s instruction strategy and to enhance the academic performance of nursing students. RCM intends to present guidance for nurse educators using the method of building concept maps for classroom and clinical settings. This study is expected to help students change their learning patterns by moving from rote learning to learning meaningful concepts, which requires students to actively engage in the learning process.

## Methods

The research design was a mixed-method (quantitative and qualitative) sequential exploratory design. This strategy is useful when developing and testing a new instrument. This approach was chosen to allow for the collection of qualitative data followed by the collection of quantitative data for analysis.

### Development of the RCM Guideline

**Figure 1:**
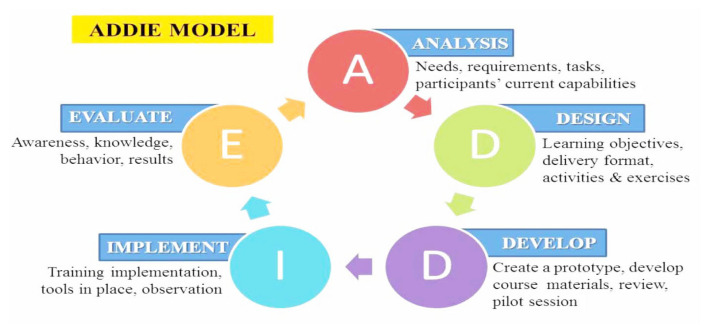
ADDIE model

**Figure 2:**
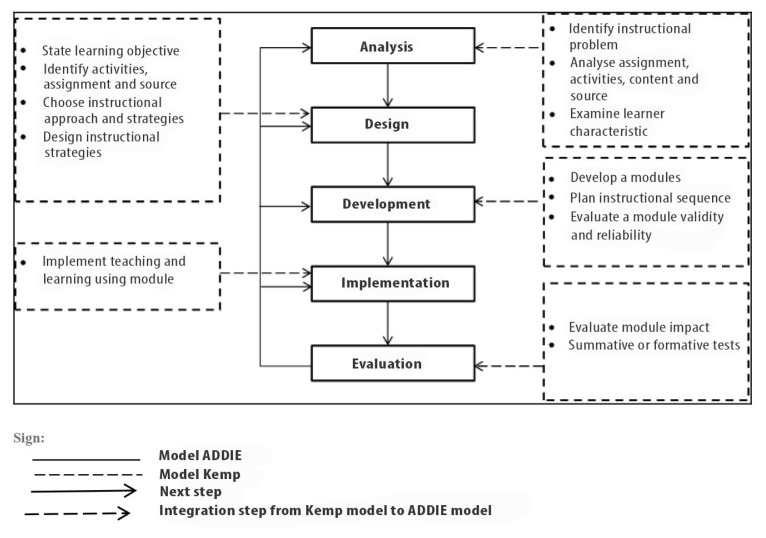
MoAFF model

**Figure 3:**
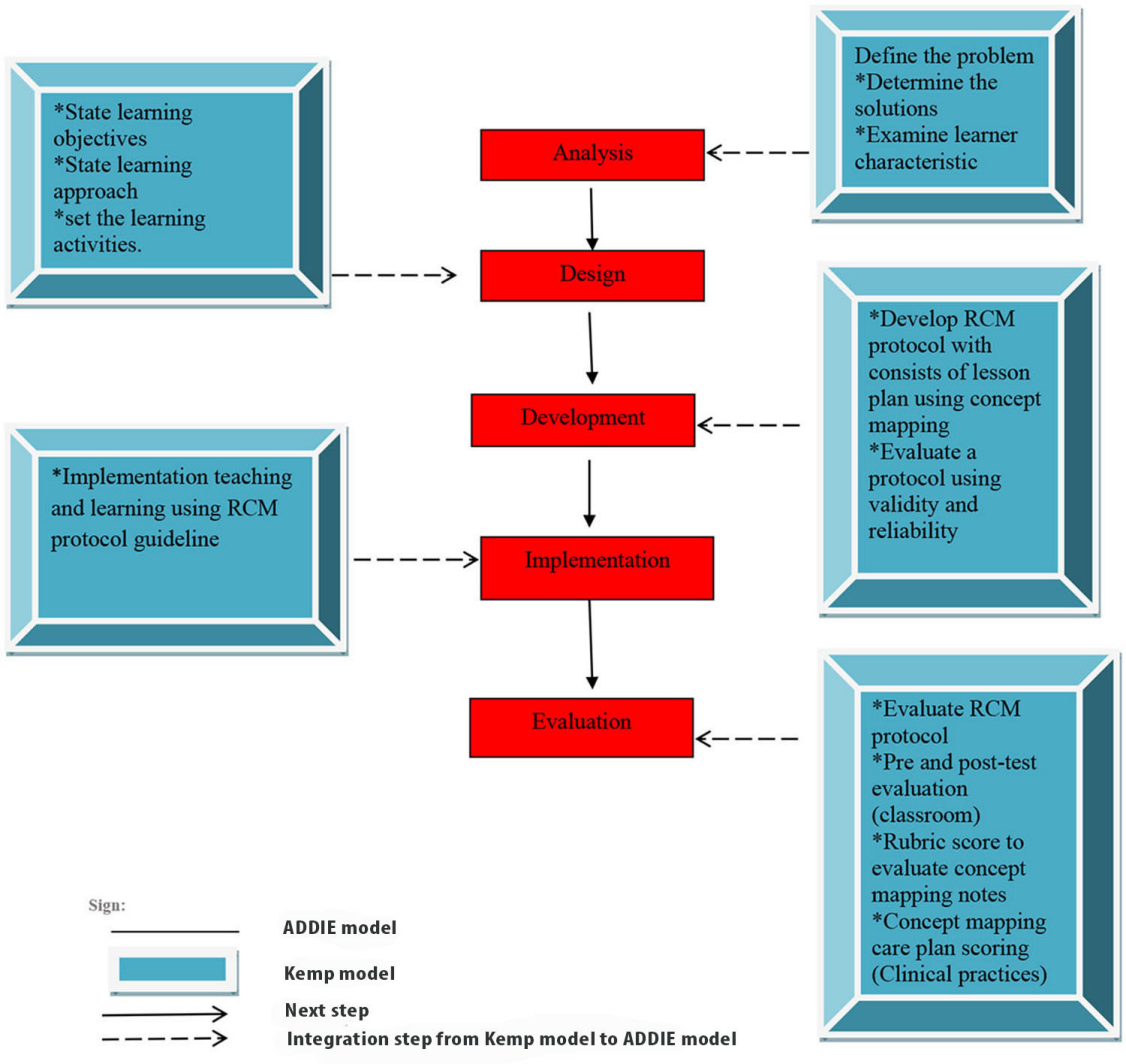
The development of RCM process adaptation from MoAFF model

**Figure 4:**
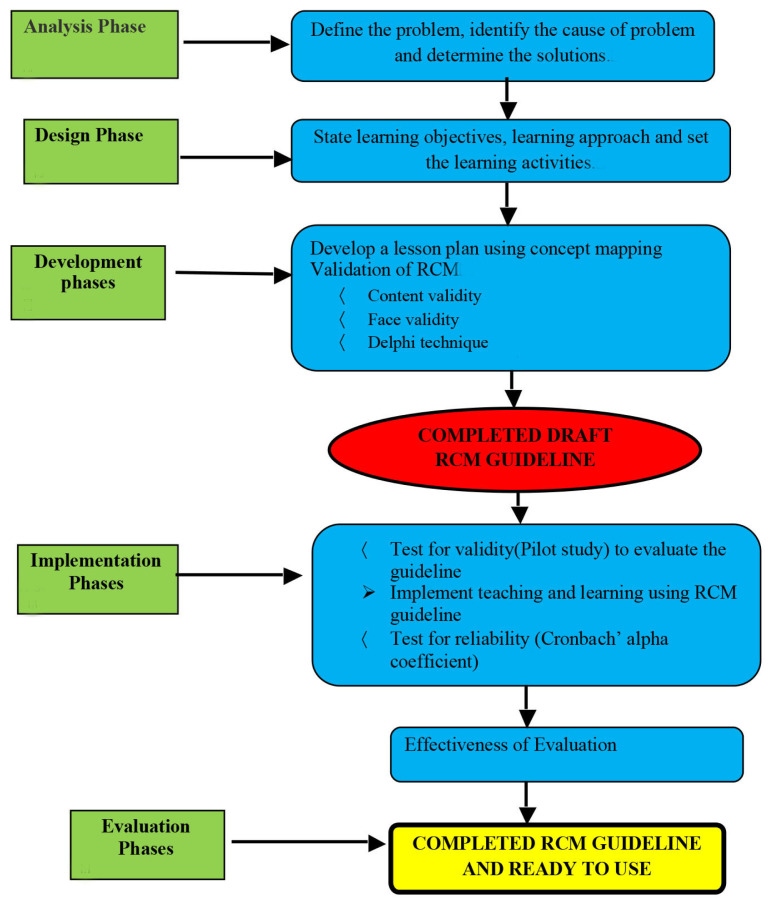
Flow chart of development of RCM guideline according to the phases of the ADDIE model

The five phases of the RCM guideline are elaborated here. Due to the intention for RCM to be used in the classroom, the guideline was also informed by the Mohd Afifi learning model (MoAFF), which adapted the Kemp model (8). The information and elements contained in the MoAFF model were easily adjusted to meet the needs of the curriculum and learning objectives of this study. Figure 1 shows the cycle of the ADDIE model. The MoAFF model is illustrated in Figure 2 and Figure 3 shows the RCM guideline adaptations from the MoAFF learning model. This section also discusses the phases of the ADDIE model and Figure 4 displays the flow chart of the development of the RCM guideline according to the ADDIE model phases.

### Analysis Phase

This is the foundation of all phases. During this phase, researchers must define the problem, identify the cause of the problem and then determine a solution. Based on observations and surveys, the researchers found that one weakness of the lecture method is that it allows students to be passive recipients of information. Students become dependent on the teacher to tell them what they need to know, and as a result, they avoid taking responsibility for their own learning. The analysis phase in this case included identifying the type of learning involved and learner characteristics such as academic background and academic achievement (as measured by the grade point average [GPA]). The analysis should reveal students’ characteristics, such as the sex ratio and the average level of students’ ability (9). Based on the model, the researchers in the present study identified the followings:

- Target group: Nursing diploma students in Semester 4 (Year 2, Semester 2) were selected because a topic being taught during the semester is related to diabetes mellitus.
- Nurse educator: Taking into account the necessary requirements for a nurse educator, four nurse educators were selected from four different nursing colleges based in various zones (eastern, northern, central and southern). The criteria for inclusion of a nurse educator was that they must have 5 years’ experience in teaching medical topics.
- Teaching topic: In this phase, the learning objectives, approach and activities are determined. The researchers determined that concept mapping should be used as the main approach to teaching the topic of medical-surgical nursing for diabetes mellitus. This topic is the primary topic of questions for the medical-surgical examination and it is a challenging subject that has reportedly been the cause of many failures.

### Design Phase

In this phase, the module is designed based on the stated learning objectives, approaches and activities. The learning objectives are set so that students will develop their concept mapping skills, creating their own concept maps regarding diabetes mellitus to enhance critical thinking and problem-solving skills. Concept mapping is used as the main approach to teaching and learning to enhance critical thinking. In that respect, the sequence in which the content is learned is critical to help students achieve the objectives (10). There are four key points of consideration when designing the module (9): i) What must students learn?; ii) How do you identify students who have learned it?; (iii) How do you assist students with learning? and finally, (iv) How can media and technology support students’ learning?

### Development Phase

**Figure 5:**
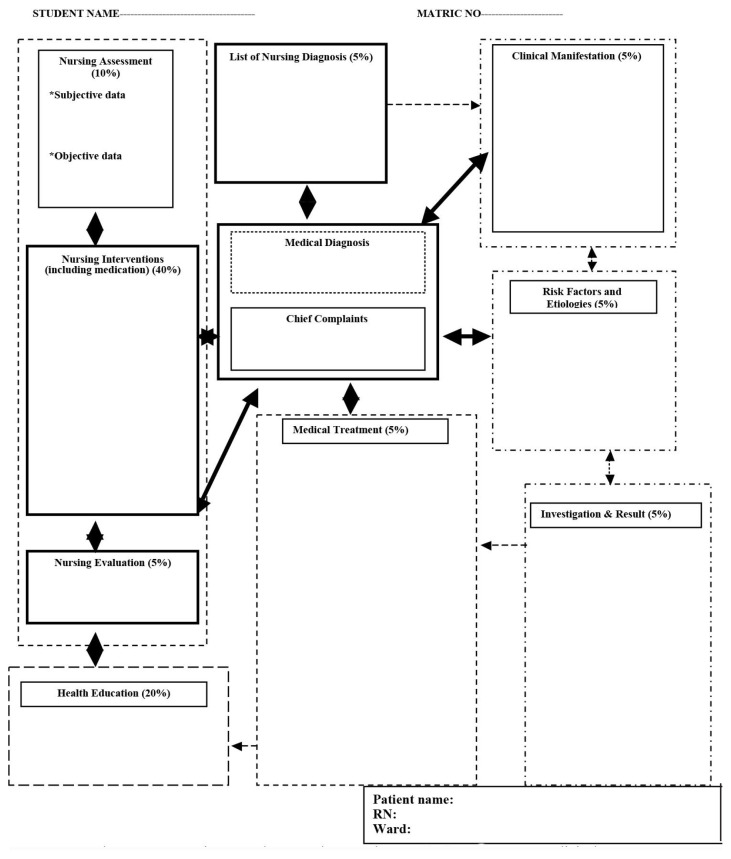
The concept mapping care plan to evaluate students’ academic performance at clinical practices

This phase is when developers build and implement the identified learning objectives. In this phase, the researchers developed a RCM comprising a lesson plan using concept mapping that comprised an assignment (a case study based on the scenario), exercise (concept mapping notes), multiple choice questions (MCQs) for student testing on what has been taught and a concept mapping care plan to evaluate students’ performance in clinical practice. The MCQs were validated with three rounds of the Delphi technique. This phase includes the validation process so that it is completed before the implementation phase. Figure 5 displays the concept mapping care plan used to evaluate students’ performance in clinical practice. This concept mapping care plan and the MCQs were validated using the Delphi technique. Rubric score evaluation was used to evaluate the concept mapping care plan adapted from Ainsley (11) to determine the pass/fail line with scores of < 33.3% (low); > 33.3%–66.6% (moderate) and > 66.6% (high).

The design used here was intended to promote a holistic view of the patient and students were instructed to do the followings:

- First, identify the patient and write their name in the box in the middle of the page where the central concept goes.
- Next, add the patient’s medical diagnosis, chief complaints and/or reason for hospitalisation.
- Working from that information, add as many relevant and related nursing diagnoses as possible in the boxes related to the main box.
- For each nursing diagnosis, list the subjective and objective data associated with the diagnosis that were identified in the case study.
- Under the relevant nursing diagnoses, list any current information about the diagnosis and the patient’s medical history, risk factors, etiologies, diagnostic tests, treatments and medications.
- List the nursing interventions that were completed in relation to the priority diagnosis.
- Next, list the nursing evaluation after all the interventions were done.
- Finally, list the nursing education that should be given to the client in relation to their health care diagnosis.

### Validation Process

The pre-implementation phase is the process of validation. Validity can be divided into four categories: i) face validity; ii) content validity; iii) criterion-related validity and iv) construct validity (12). In this study, two types of validity were used: i) content and ii) face validity.

### Validation of Concept Mapping Care Plan and MCQs

The Delphi technique was used to collect the data for validating the protocol (13, 14). The Delphi technique is a commonly used data collection process aimed at achieving expert agreement about the content being evaluated (15). It is important to note that the selection of panels within a certain time frame when conducting a Delphi’s study must be done in a careful manner so that the experts selected for the panels represent the two areas of the subject matter and the pedagogy. This is because cooperation from participants in the Delphi method is key to successful implementation. Investigators must play an active role during implementation to ensure positive responses (15).

In this study, the researchers chose the Delphi research method to gain consensus on both the concept mapping care plans and the MCQs by putting together a panel composed of 10 experts from among faculties at Universiti Sains Malaysia (USM), nursing lecturers at USM and nursing tutors from Kubang Kerian Nursing College. With this technique, the experts were given the opportunity to consider the biases and objections of other panel members in an environment free of bias and where they were free to express their opinions. In the first round, the panel was given the structured questionnaire regarding items on the concept mapping care plan and the MCQs about diabetes mellitus. In the second round, the mean and median values from the results of round 1 were added (and in the third round, the mean and median values of round 2 were added). The panelists were asked to rate the categorised responses from round 1 on a scale of 1 to 5, with 1 being very irrelevant and 5 being very relevant. This feedback process encourages the Delphi panelists to reassess their initial judgments about the information provided in previous iterations.

Data were analysed to check for consistency in the experts’ responses between rounds. An instrument developed based on the Delphi technique research findings was also examined and validation was obtained from the experts with regard to content and face validity. The analysis of the consensus data from the experts was based on the median, interquartile range and quartile deviation (QD) from the data of rounds 1, 2, and 3. In the validation process, the researcher used these three rounds of the Delphi method to validate the 20 MCQ items and the 9 concept mapping care plan items. In this study, the researcher used the formula from Norizan (16) as a guideline for reaching consensus from the panel on the validity and the importance of the items (Table 1). The formula used to identify the QD was as follows:

(1) $$\begin{array}{l} \text{Q}=\frac{\text{Interquartile range}}{2}\\ =\frac{\left({\text{Q}}_{3}-{\text{Q}}_{1}\right)}{2} \end{array}$$

The concept mapping care plan and MCQ questions were ultimately found to meet consensus when all statements received a median consensus greater than 4, implying that the level of importance of the statements was high. If the median value had been less than 3.5, the importance of the statements would have been low. The Delphi technique is an accepted method for gathering data from experts in the domain under study and it is an effective process for allowing a group of individuals to work together as a whole when solving a complex problem.

### Validation of RCM Content

As already noted, two types of validity assessments were carried out: i) content validity and ii) face validity.

#### i) Content validity

Content validity refers to the extent to which the content can be used as an effective measure of the item being reviewed or studied (17). It is used to determine the instrument’s representativeness of the content. Content validity has been defined as “the degree to which an instrument has an appropriate sample of items for the construct being measured” (18). A range of 3 to 10 is the number of experts recommended by studies in the literature to have an adequate review panel and content validation (19, 20).

Content validity for this lesson plan was established using the review of the seven expert panelists. For checking the validity of the RCM guideline, there were four evaluators: one expert in the field of nursing education (Vice-principal, PSG College of Nursing, Coimbatore, Tamilnadu, India) and one expert in teaching pedagogy from USM, along with two lecturers from the Institute of Teacher Education (IPG) Kota Bharu and IPG Jitra, Kedah. The experts were contacted through personal phone calls or emails to ask for their willingness. Later, an official letter of appointment printed on university letterhead was sent to those who accepted the invitation. In the review process, experts were asked to read items based on the content domain and then judge the item’s relevance on a 5-point scale (1 = strongly disagree; 2 = disagree; 3 = neither agree nor disagree; 4 = agree, and 5 = strongly agree). The items included the suitability of the target group, the suitability of time in conjunction with the objectives and procedures in an activity, whether the protocol content would be able to improve the levels of participants’ academic and personal achievements, and whether it would be able to change participants’ attitudes on excellence.

To validate the content of the diabetes mellitus topic, the researchers appointed three evaluators — two in the field of nursing education and one experts in teaching pedagogy from USM whose knowledge is considered ample and respectable. In the review process, the experts were asked to judge the relevance of the 10 items based on the relevancy, accuracy, and sufficiency according to a 5-point scale (1 = strongly disagree; 2 = disagree; 3 = neither agree nor disagree; 4 = agree, and 5 = strongly agree).

#### ii) Face validity

Face validity refers to the assessment of any measuring instrument in terms of its relevance and if it can provide suitable, unambiguous and clear measurements (21). Several aspects needed to be assessed to determine the face validity in this study, including spelling, spacing between words, font size, instructions, and the format used (21). The first process was achieved with an expert’s assessment of the first draft of the instruments.

### Calculating the Content Validity Score

Upon receiving the feedback of the four expert panelists, the researcher analysed the content validity using the formula produced by Sidek Mohd and Ahmad (22). The result is in the form of a percentage (%). This formula reported good content validity with an achievement score meeting the benchmark of greater than 70%. If the result is less than 70%, it is advisable to recheck the content according to the objective of the study. This formula is displayed as Equation (2):

(2) $$\text{Content validity achievement}=\frac{\text{Total score from expert}}{\text{Maximum score }(25)}\times 100$$

### Implementation Phase (Pilot Study)

An official letter was sent to the director of the Kubang Kerian Nursing College to obtain permission to conduct a pilot study, which was conducted with 30 respondents from the student body in Semester 4. The aim was to develop and test the adequacy of the research instruments. To establish internal validity, pre-test and post-test instruments were employed—the pre-test was used so the researcher could investigate whether the concept mapping strategies ‘improved’ the students’ test scores. The implementation stage reflects the continuous modification of the program to ensure that maximum efficiency and positive results are obtained. In this phase, the researchers also provided a briefing and guidance to the nurse educator regarding the RCM guideline so that the nurse educator would be able to use it effectively.

### Evaluation Phase

The aim of this phase was to check the reliability of the RCM guideline. This phase involves the testing of the developed assessment to ensure that it achieved the learning objectives. The analysis measured the RCM guideline at 0.816 for 10 items. The most common methods of measurement used were test–retest reliability, alternative form reliability and internal consistency reliability (23). One way to assess the reliability index of a module is by using Cronbach’s alpha (24), which is the most widely used method of generating internal consistency coefficients to establish reliability. In this study, the researchers used Cronbach’s alpha for testing the measurement instruments and RCM guideline. There are no fixed standards either locally or internationally for the best coefficient reliability value, but reliability is generally considered good when the index value is greater than 0.70 (17, 25).

## Results

### Delphi Technique Validation of Concept Mapping Care Plan and MCQs

In the validation process, consensus on the concept mapping care plan for this study after the first, second and third Delphi rounds was reached when the QD of each statement was less than or equal to 0.5 (QD ≤ 0.5). This validation indicated a high level of consensus. Tables 2 and 3 display the experts’ consensus data based on median, interquartile and QD from rounds 1, 2, and 3. The concept mapping care plan and MCQs were found to meet consensus when all statements received a median consensus greater than 4.

### Validation of RCM Content

#### Results of Content Validity Analysis of Lesson Plan on Diabetes Mellitus

For this content validity, the researchers divided the measure of the questions into three categories: i) relevancy; ii) accuracy and iii) sufficiency. In the review process, experts were asked to read and assess each item in terms of its relevancy to the topic, its accuracy on the topic, and its sufficiency to increase student achievement, rating each on a 5-point scale (1 = strongly disagree; 2 = disagree; 3 = neither agree nor disagree; 4 = agree and 5 = strongly agree). In Part 1 (relevancy assessment), the highest ratings were given by Evaluators 1 and 2 (100%), followed by Evaluator 3 (80%). In Part 2 (accuracy assessment), Evaluators 1 and 3 gave ratings of 100%, followed by Evaluator 2, with 72%. The ratings for Part 3 (sufficiency) are 96% by Evaluator 1, 70% by Evaluator 2 and 80% by Evaluator 3. Overall, the evaluators agreed that the RCM guideline was relevant, accurate and sufficient for teaching the topic diabetes mellitus. Validity was assessed for the items on the questionnaire using a 5-point Likert scale and the items reached mean values of 5 and 4.

## Results of Validation Analysis of RCM Guideline Content

Table 4 shows that the highest score for this validation was given by Evaluator 4 (96%), followed by Evaluator 3 (92%) and Evaluator 2 (84%), while the lowest was given by Evaluator 1 (72%). Overall, the evaluators agreed that the RCM was related to the objective of teaching and was suitable for the target group. Validity was assessed for the questionnaire items using a 5-point Likert scale where the mean values were 5 and 4. The content validity index (CVI) scores ranged from 0.72 to 0.96.

The information in Table 5 shows that the entire panel of evaluators agreed that the RCM was suitable for the target group (100%). For the content validity question, the panel agreed that the content of this guideline can be properly implemented, with two evaluators (50%) choosing ‘strongly agree’ and the other two evaluators (50%) choosing ‘agree.’ In terms of the RCM care plan’s appropriateness of time with the objectives and procedures, ability to enhance academic and individual accomplishments, and ability to help change behaviours toward striving for excellence, one evaluator (25%) chose ‘strongly agree,’ two evaluators (50%) chose ‘agree,’ and the remainder (25%) chose ‘neither agree nor disagree.’

### Content Validity

In this study, the content was considered valid when it would meet the needs of the target population, the implementation of the module is considered satisfactory, it is used for a sufficient time, it can increase student achievements and it can prompt students to endeavour for excellence (22). The majority of the panelists agreed that the content domains were well represented in the RCM guideline. The CVI was more than 72%, which means that this RCM guideline has high levels of both reliability and validity.

### Reliability of the RCM Guideline

A validity coefficient of 0.70 or greater means the test instrument’s measures are acceptable for the stated purpose of providing good measurements (26). A minimum reliability index value of 0.50 is acceptable for a module’s reliability index value (25). The results here indicate that the RCM guideline was applicable for nursing students and was a good and innovative teaching method for enhancing the academic performance of nursing students.

## Discussion

The development of the RCM guideline differed from other studies using concept mapping as a teaching method in the way that it was developed according to the phases of the ADDIE model. Each phase is explained in detail and the design modules are based on the MoAFF model, which is well suited for the approach of using concept mapping teaching methods for all the activities in the building and is student-centred for learning and places the educator as facilitator. This model’s features of flexibility and nonlinearity allow researchers to easily adapt and combine it, and the information and elements can be adjusted and modified to meet the needs of the curriculum and its learning objectives. The integration of this model with the Kemp model adds convenience because it is oriented to teaching in the classroom. In addition, RCM is one of the teaching methods that specifies guidelines for nurse educators to harmonize the classroom and clinical settings, while most previous studies relied solely on classroom teaching (27–30).

With the aid of rubric scores to evaluate mapping care plan concepts in clinical practice, it allows students to analyse, assess and evaluate data that are in line with patient care. Via concept mapping, the care plan encourages nursing students to think more independently and gives them more confidence to practice in the clinical area, because all the respondents aspire to find and give personal nursing care to their patients. This instruction enhancement will change students from passive learners into active learners during the clinical setting. Researchers believe that concept mapping care plans can be implemented as a replacement for nursing processes that have already been practiced in the clinical setting. Concept mapping based on the nursing process was more appropriate to nursing as a practical field and was used in the construction of clinical experience (31).

Hence, RCM can help nurse educators with their teaching techniques, such as in providing ideas for planning, organising and a teaching plan that can provide references for their evaluation of student learning. RCM can be an alternative teaching and learning method for delivering effective learning using concept mapping. This study’s results imply that nurse educators should incorporate concept mapping in their teaching methods to improve critical thinking of students and provide an in-depth approach to learning (32). Without an effective learning strategy, students will still learn something, but what they learn will be easily forgotten (33).

## Conclusion

The reliability of the RCM guideline was 0.816. This indicates that the RCM guideline was pertinent for nursing students and will assist nurse educators in using teaching methods based on concept mapping. Indirectly, it will help to ease the teachers’ burden of designing active learning projects for students. RCM is beneficial in theoretical knowledge and practical skills. Learning activities using the RCM guideline are more student-centered and the teacher acts as a facilitator. Researchers are of the view that there is a need for changes in nursing curriculum, such as implementing student-centred learning patterns in which students are helped to understand the concept rather than merely memorizing facts. The researcher recommends that all allied health educators should officially include a concept mapping strategy in their teaching methods.

## Figures and Tables

**Table 1:** Level of consensus and importance

Quartile deviation (QD)	Level of consensus	Median	Level of Importance
Less or equal to 0.5 (QD ≤ 0.5)	High	4 and above (M ≥ 4)	High
More than 0.5 and less than or equal to 1.0 (0.5 ≤ QD ≤ 1.0)	Moderate	3.5 and less (M ≤ 3.5)	Low
More than or equal to 1.0 (QD ≥ 1.0)	Low and no consensus	-	-

Note: Formula by Norizan (16) on classifications of consensus was determined at three levels

**Table 2:** Consensus in concept mapping care plan through three rounds of Delphi technique

Item	Rounds of Delphi								
	Round 1			Round 2			Round 3		
	Median	Mean	QD	Median	Mean	QD	Median	Mean	QD
Statement 1	5.0	4.7	0.5	5.0	4.7	0.5	5.0	4.7	0.5
Statement 2	5.0	4.7	0.5	5.0	4.7	0.5	5.0	4.7	0.5
Statement 3	5.0	5.0	0	5.0	4.7	0.5	5.0	4.7	0.5
Statement 4	5.0	5.0	0	5.0	4.4	1.0	5.0	4.7	0.5
Statement 5	5.0	5.0	0	5.0	4.4	1.0	5.0	4.7	0.5
Statement 6	5.0	5.0	0	5.0	4.7	0.5	5.0	4.7	0.5
Statement 7	5.0	4.7	0.5	5.0	4.1	1.5	5.0	4.7	0.5
Statement 8	5.0	5.0	0	5.0	4.7	0.5	5.0	4.7	0.5
Statement 9	5.0	5.0	0	5.0	4.7	0.5	5.0	4.7	0.5

**Table 3:** Consensus in multiple choice questions (MCQ) through three rounds of Delphi technique

Item	Rounds of Delphi								

	Round 1			Round 2			Round 3		
	Median	Mean	QD	Median	Mean	QD	Median	Mean	QD
Statement 1	4.0	3.5	2.0	4.0	4.0	1.0	5.0	4.6	0.5
Statement 2	4.0	3.8	1.5	4.0	4.0	1.0	4.5	4.5	0.5
Statement 3	4.0	3.8	1.5	4.0	4.3	0.5	4.5	4.5	0.5
Statement 4	4.0	4.4	0.5	3.0	3.8	1.0	4.0	4.4	0.5
Statement 5	4.0	4.1	1.0	5.0	4.4	1.0	5.0	4.6	0.5
Statement 6	4.0	3.5	2.0	4.0	3.7	0.5	5.0	4.8	0.5
Statement 7	4.0	3.8	1.5	3.0	3.4	0.5	4.5	4.7	0.5
Statement 8	4.0	3.8	1.5	4.0	3.7	0.5	4.5	4.6	0.5
Statement 9	4.0	4.1	1.0	5.0	4.4	1.0	5.0	4.7	0.5
Statement 10	4.0	4.1	1.0	5.0	4.4	1.0	4.5	4.5	0.5
Statement 11	4.0	4.0	0	4.0	4.4	0.5	5.0	4.8	0.5
Statement 12	5.0	4.7	0.5	0	4.1	1.0	4.5	4.5	0.5
Statement 13	3.0	3.3	0.5	5.0	4.7	0.5	4.0	4.4	0.5
Statement 14	4.0	4.0	0	5.0	4.4	1.0	4.5	4.5	0.5
Statement 15	3.0	3.3	0.5	4.0	4.4	0.5	4.0	4.3	0.5
Statement 16	4.0	4.4	0.5	5.0	4.4	1.0	5.0	4.6	0.5
Statement 17	4.0	3.5	2.0	4.0	3.4	1.0	4.0	4.1	1.0
Statement 18	4.0	3.5	2.0	4.0	4.0	0	4.0	4.4	0.5
Statement 19	4.0	3.8	1.5	4.0	3.7	0.5	4.5	4.5	0.5
Statement 20	3.0	3.2	2.0	4.0	4.0	0	4.0	4.2	1.0

**Table 4:** Division of validity achievement percentage of four experts’ evaluator

RCM	Expert’s Score (x/25) × 100	Total of validity achievement (%)	Validity index
Evaluator 1	18/25 × 100	72	0.72
Evaluator 2	21/25 × 100	84	0.84
Evaluator 3	23/25 × 100	92	0.92
Evaluator 4	24/25 × 100	96	0.96

**Table 5:** Content validation process of the RCM for lesson plan instrument by four expert evaluators

Content validity question	Scale				
	1 Strongly disagree *n* (%)	2 Disagree *n* (%)	3 Neither agree or disagree *n* (%)	4 Agree *n* (%)	5 Strongly agree *n* (%)
i) The suitability of the target group					4 (100)
ii) The content of this RCM can be implemented properly				2 (50)	2 (50)
iii) The suitability of time with the objectives and procedures in an activity			1 (25)	2 (50)	1 (25)
iv) The RCM content is able to improve participants’ academic and personal achievement			1 (25)	2 (50)	1 (25)
v) The RCM content is able to help change participants’ attitude towards excellence			1 (25)	2 (50)	1 (25)

## References

[1] Hicks-Moore S (2005). Clinical concept maps in nursing education: an effective way to link theory and practice. Nurs Educ Pract.

[2] Conceição SCO, Taylor LD (2007). Using a constructivist approach with online concept maps: Relationship between theory and nursing education. Nurs Educ Perspect.

[3] Hsu LL (2006). An analysis of clinical teacher behaviour in a nursing practicum in Taiwan. J Clin Nurs.

[4] Chen S, Liang T, Lee M, Liao I (2011). Effects of concept map teaching on students’ critical thinking and approach to learning and studying. J Nurs Educ.

[5] Morrison GR, Ross SM, Kalman HK, Kemp JE (2010). Designing effective instruction.

[6] Rossett A (1987). Training needs assessment.

[7] Gustafson KL, Branch RM, Reiser RA, Dempsey JV (2002). What is instructional design?. Trends and issues in instructional design and technology.

[8] Yusop H (2001). Reka Bentuk dan Sistem Instruksi.

[9] Shambaugh NN, Magliaro SG (2006). Instructional design: A systematic approach for reflective practice.

[10] Kemp JE, Morrison GR, Ross SM (1998). Designing effective instruction.

[11] Ainsley J CEO analysis: ten years of analysis of the course experience questionnaire surveys.

[12] Sekaran U (2003). Research methods for business: A skill building approach.

[13] Dalkey NC (1969). The Delphi method: an experimental study of group opinion.

[14] Wilhelm WJ (2001). Alchemy of the oracle: the Delphi technique. The Delta Pi Epsilon Journal.

[15] Ab Latif R, Mohamed R, Dahlan A, Mat Nor MZ (2016). Using Delphi technique: making sense of consensus in concept mapping structure and multiple choice questions (MCQ). Educ Med J.

[16] Norizan AR (2003). Computer competency of in-service ESL teachers in Malaysian secondary schools. PhD diss.

[17] Chua YP (2006). Asas statistik penyelidikan.

[18] Polit DF, Beck CT (2004). Nursing research: principles and methods.

[19] Grant JS, Davis LL (1997). Selection and use of content experts in instrument development. Res Nurs Health.

[20] Rubio DM, Berg-Weger M, Tebb SS, Lee ES, Rauch S (2003). Objectifying content validity: Conducting a content validity study in social work research. Soc Work Res.

[21] Oluwatayo JA (2012). Validity and reliability issues in educational research. J Educational Soc Res.

[22] Sidek Mohd N, Ahmad J (2005). Pembinaan modul: bagaimana membina modul latihan dan modul akademik.

[23] Netemeyer RG, Bearden WO, Sharma S (2003). Scaling procedures: issues and applications.

[24] Ebel RL, Frisbie DA (1991). Essentials of educational measurement.

[25] Jackson SL (2006). Research methods and statistics: A critical thinking approach.

[26] George D, Mallery P (2001). SPSS for Windows step by step: a simple guide and reference 100 update.

[27] Hsu L, Hsieh S (2005). Concept maps as an assessment tool in a nursing course. J Prof Nurs.

[28] Novak JD (2007). Concept maps. What the heck is this?.

[29] Novak JD, Cañas AJ (2008). The theory underlying concept maps and how to construct and use them.

[30] Moattari M, Soleimani S, Moghaddam NJ, Mehbodi F (2014). Clinical concept mapping: does it improve discipline-based critical thinking of nursing students?. Iran J Nurs Midwifery Res.

[31] Chabeli MM (2010). Concept-mapping as a teaching method to facilitate critical thinking in nursing education: a review of the literature. Health SA.

[32] Jamaludin R (2003). Multimedia dalam pendidikan.

